# Management of Nephrolithiasis in Pregnancy: Multi-Disciplinary Guidelines From an Academic Medical Center

**DOI:** 10.3389/fsurg.2021.796876

**Published:** 2021-12-22

**Authors:** Matthew S. Lee, Michael A. Fenstermaker, Emily E. Naoum, Suzanne Chong, Cosmas J. Van de Ven, Melissa E. Bauer, Joanna A. Kountanis, James H. Ellis, James Shields, Sapan Ambani, Amy E. Krambeck, William W. Roberts, Khurshid R. Ghani

**Affiliations:** ^1^Department of Urology, University of Michigan, Ann Arbor, MI, United States; ^2^Department of Urology, Northwestern University Feinberg School of Medicine, Chicago, IL, United States; ^3^Kaiser Permanente Group, Department of Urology, Washington, DC, United States; ^4^Department of Anesthesia, Critical Care and Pain Medicine, Massachusetts General Hospital, Boston, MA, United States; ^5^Department of Radiology, Indiana University, Indianapolis, IN, United States; ^6^Department of Obstetrics and Gynecology, University of Michigan, Ann Arbor, MI, United States; ^7^Department of Anesthesiology, Duke University, Durham, NC, United States; ^8^Department of Anesthesiology, University of Michigan, Ann Arbor, MI, United States; ^9^Department of Radiology, University of Michigan, Ann Arbor, MI, United States; ^10^Department of Interventional Radiology, University of Michigan, Ann Arbor, MI, United States

**Keywords:** pregnancy, kidney stones, nephrolithiasis, obstetric, ureteroscopy

## Abstract

**Introduction:** The management of nephrolithiasis during pregnancy can be stressful for urologists due to concerns for investigations and treatments that may pose risk of fetal harm, and unfamiliarity with optimal management of these complex patients. In response, we created multi-disciplinary evidence-based guidelines to standardize the care for obstetric patients presenting with flank pain and suspicion for nephrolithiasis.

**Methods:** A multi-disciplinary team involving Urology, Obstetric Anesthesiology, Obstetrics and Gynecology, Diagnostic Radiology, and Interventional Radiology from a single academic medical center was assembled. A PubMed search was performed using keywords of pregnancy/antepartum, nephrolithiasis/calculi/kidney stones, ureteroscopy, non-obstetric surgery, complications, preterm delivery, MRI, computerized tomography, renal bladder ultrasound (RBUS), and anesthesia to identify relevant articles. Team members reviewed their respective areas to create a comprehensive set of guidelines. One invited external expert reviewed the guidelines for validation purposes.

**Results:** A total of 54 articles were reviewed for evidence synthesis. Four guideline statements were constructed to guide diagnosis and imaging, and seven statements to guide intervention. Guidelines were then used to create a diagnostic and intervention flowchart for ease of use. In summary, RBUS should be the initial diagnostic study. If diagnostic uncertainty still exists, a non-contrast CT scan should be obtained. For obstetric patients presenting with a septic obstructing stone, urgent decompression should be achieved. We recommend ureteral stent placement as the preferred intervention if local factors allow.

**Conclusions:** We present a standardized care pathway for the management of nephrolithiasis during pregnancy. Our aim is to standardize and simplify the clinical management of these complex scenarios for urologists.

## Introduction

Nephrolithiasis is the most common non-obstetric indication for hospital admission in obstetric patients (1). It is estimated to occur in 1:200 to 1:1,500 pregnancies and can cause complications for the fetus and the mother including: pre-term labor, premature delivery, infectious complications, loss of kidney, or even loss of the fetus (1–3). Due to the potential negative outcomes for the mother and fetus and fears of litigation, management of nephrolithiasis during pregnancy can be anxiety provoking for urologists.

During pregnancy there is a physiologic increase in the glomerular filtration rate as well as elevated 1, 25 Dihydroxy Vitamin D levels. These two factors result in hypercalciuria (4) and rapid encrustation of ureteral stents or percutaneous nephrostomy tubes that may be placed for obstructing upper urinary tract stones. The high rate of encrustation necessitates frequent tube exchanges and repeated exposures to anesthesia (5, 6), which carries risks of aspiration, hypotension, and pre-term labor to the mother and fetus (7). Thus, there has been increased interest in performing primary ureteroscopy, which would avoid indwelling tubes and the need for repeat anesthetic exposures.

Due to concerns of radiation exposure to the fetus, renal bladder ultrasound (RBUS) is the diagnostic modality of choice during pregnancy (8). However, during pregnancy there is a physiologic hydronephrosis that occurs secondary to progesterone induced smooth muscle relaxation and compression of the ureter by the gravid uterus (5), which reduces the diagnostic utility of RBUS. While non-contrast computed tomography (NC-CT) of the abdomen/pelvis is the gold standard for diagnosing urinary stones and can safely be obtained during pregnancy (9), many urologists are apprehensive to order tests using ionizing radiation due to fear of litigation. Thus, diagnosis of nephrolithiasis during pregnancy can be difficult.

In our institution the on-call management of the obstetric patient with acute flank pain and suspected renal colic was determined to be variable, with concern among faculty as how to manage these patients appropriately in a stepwise fashion. As a result, a multi-disciplinary team assembled with the goal to create evidence-based, comprehensive set of guidelines to guide urologists in the management of nephrolithiasis during pregnancy.

## Methods

We performed a comprehensive PubMed? search for articles published in the English language from 1990 to 2020 using keywords: pregnancy/antepartum, nephrolithiasis/calculi/kidney stones, ureteroscopy, non-obstetric surgery, complications, preterm delivery, MRI, computerized tomography, renal bladder ultrasound (RBUS), and anesthesia to identify relevant original research articles and reviews. Additional publications were identified by reviewing the reference lists of pertinent articles identified on the initial literature search. A multi-disciplinary team involving Urology, Obstetric Anesthesiology, Obstetrics and Gynecology, Diagnostic Radiology, and Interventional Radiology was assembled. Individuals of our multi-disciplinary team reviewed their areas of expertise. A total of 54 articles (listed in Appendix A) were reviewed in detail and 47 were included in the formation of these guidelines (shown in the references).

## Results

A total of 11 guideline statements were constructed; four to guide diagnosis and imaging, and seven to guide intervention.

### Part 1: Initial Work Up/Imaging

#### Recommendation 1

The management of the gravid patient with suspected symptomatic nephrolithiasis should emphasize a multidisciplinary approach, with early involvement of obstetrics, radiology, and anesthesiology teams. The on-call urology team and the on-call obstetrics (OB) team should also be notified when the patient first presents. The OB service can make recommendations regarding need for deep vein thrombosis (DVT) prophylaxis while patients are admitted (10). *Clinical principle*.

#### Recommendation 2

**Figure 1:**
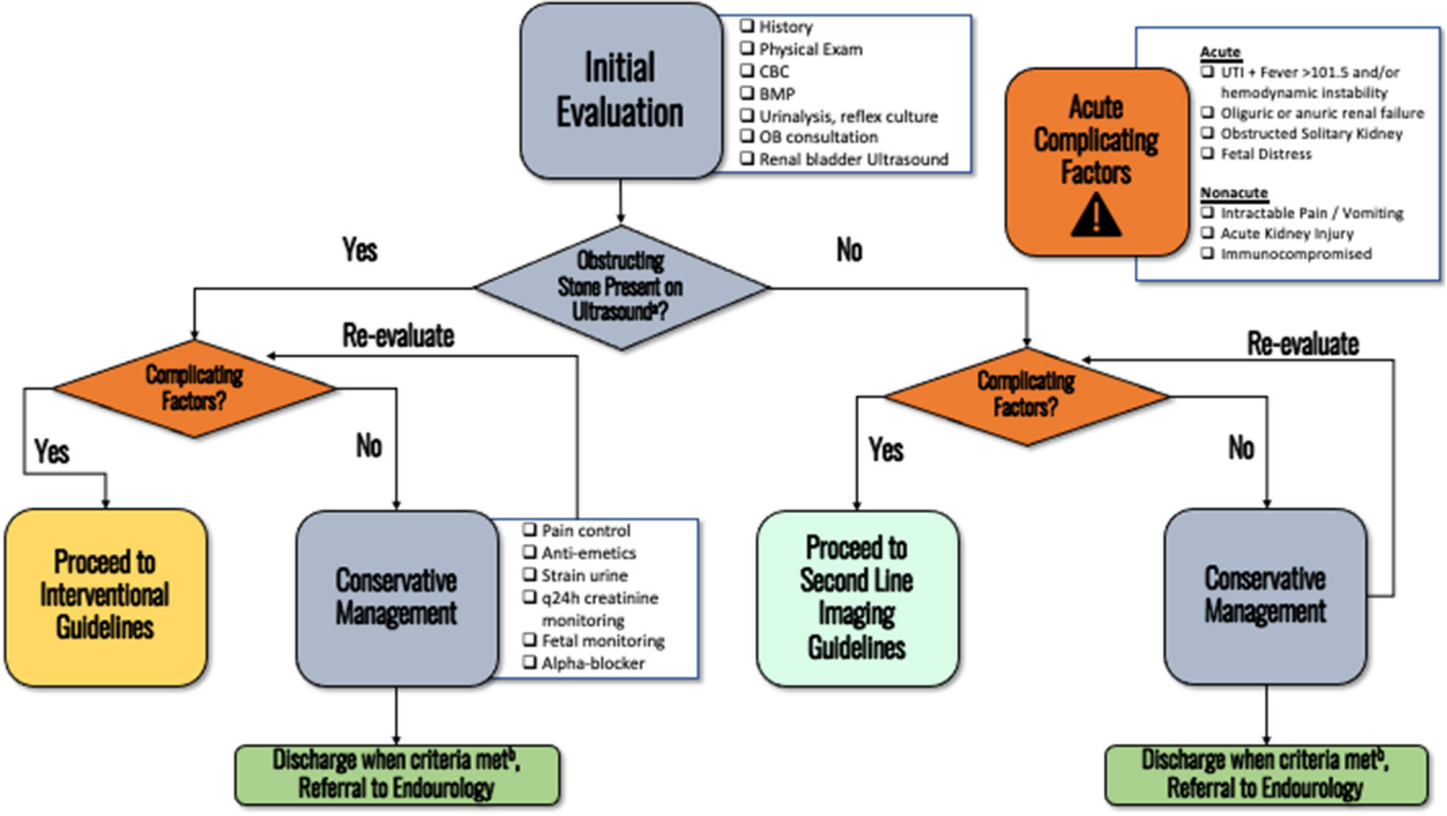
The initial evaluation of an obstetric patients presenting with nephrolithiasis. **(a)** Pain is adequately controlled on oral pain medications, nausea well controlled and able to eat/drink, no AKI and stable creatinine, afebrile, non-concerning fetal monitoring. **(b)** When more emergent diagnosis is not needed. Consider performing doppler for RI, ureteral jets, or transvaginal ultrasonography (if distal stone suspected) if not already performed on initial ultrasound.

Initial evaluation should include patient history, relevant obstetric and pregnancy history, physical exam, urinalysis with reflex urine culture, basic metabolic panel, and complete blood count (Figure 1). Fetal monitoring may be initiated based on gestational age as determined by the OB service. *Clinical principle*.

#### Recommendation 3

RBUS should be the first line imaging modality for obstetric patients presenting with renal colic suspicious for obstructing nephrolithiasis. Elements of a high-quality report include renal indices and evaluation of ureteral jets. Transvaginal ultrasound can be considered for more accurate imaging of distal ureteral stones. *Strong recommendation, Evidence Strength A*.

#### Recommendation 4

If the diagnosis of obstructing nephrolithiasis remains uncertain and there is a change in clinical status of the patient that would otherwise necessitate interventional management, second line imaging should be offered.

In the acute clinical setting (fever, hypotension, or considering intervention for intractable symptoms), a low dose non-contrast CT should be obtained. *Strong recommendation, Evidence Strength A*.

**Figure 2:**
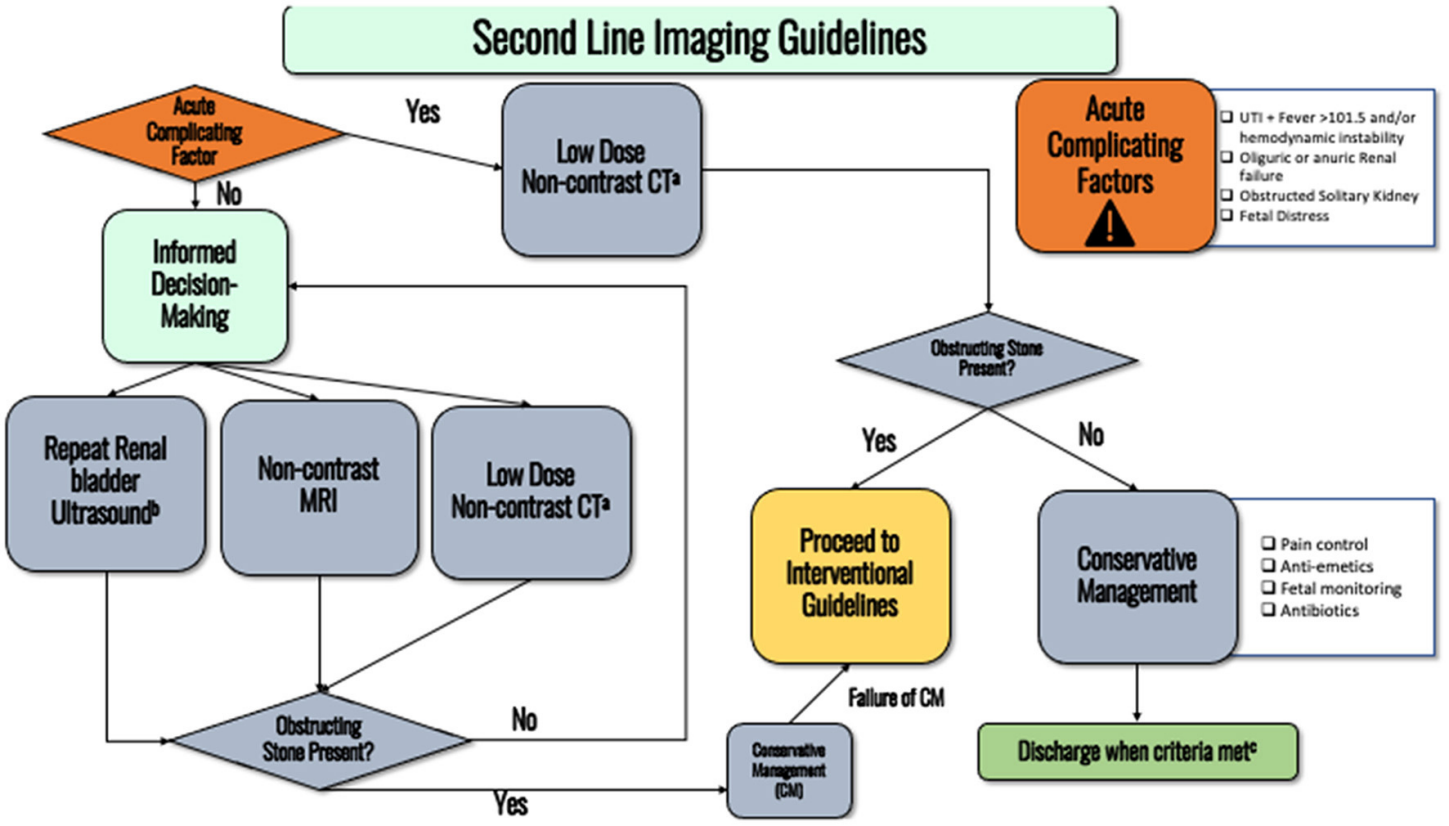
Flowchart detailing the second-line imaging guidelines. **(a)** Consider discussing with medical physicist to ensure lowest possible radiation dose is administered. **(b)** Pain is adequately controlled on oral pain medications, nausea well controlled and able to eat/drink, no AKI and stable creatinine, afebrile, non-concerning fetal monitoring. **(c)** Transvaginal ultrasound can more accurately detect distal ureteral stones.

In the non-acute setting, repeat RBUS, non-contrast MRI with HASTE, or non-contrast CT (NC-CT) should be discussed as second-line options (see discussion–Figure 2). Ultimately, the next choice of imaging modality should be based on shared decision making with the patient as there are risks and benefits to each, as discussed below. If the patient has already been exposed to multiple irradiating studies throughout the pregnancy, consultation with a medical physicist from radiology (if available) to help inform the clinical decision should be considered. *Conditional recommendation, Evidence Strength C*.

### Part 2: Intervention

#### Recommendation 5

If a patient's symptoms can be managed with analgesics and there are no complicating factors, a trial of passage with hydration and analgesia is warranted. Medical expulsive therapy appears to be safe (Figure 1) (11). Patients failing medical expulse therapy should follow up with the Urologist to discuss ureteroscopy. *Strong recommendation, Evidence Strength B*.

#### Recommendation 6

**Figure 3:**
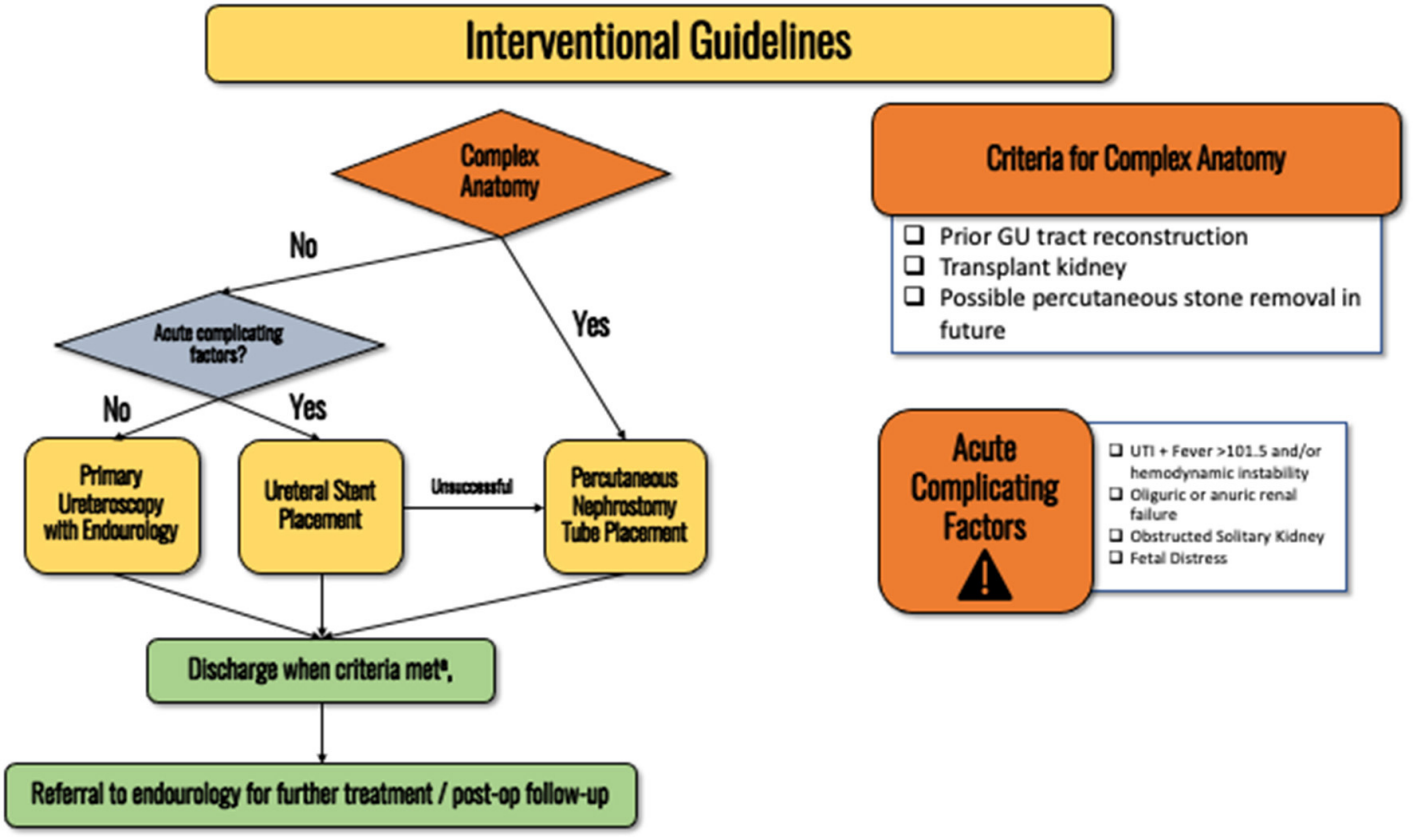
Flowchart detailing the interventional guidelines for obstetric patients with nephrolithiasis. **(a)** Pain is adequately controlled on oral pain medications, nausea well controlled and able to eat/drink, no AKI and stable creatinine, afebrile, non-concerning fetal monitoring.

If there is **concern for a septic obstructing stone, urgent collecting system decompression is required with ureteral stent placement**, this recommendation holds regardless of gestational age (see discussion–Figure 3). Stenting is safer for the patient and the fetus given that percutaneous nephrostomy (PCN) placement would require prone positioning (difficult to access airway and perform fetal monitoring). Furthermore, obstetric patients are a high aspiration risk due to mechanical changes from the gravid uterus and the effects of progesterone including impaired gastric motility and lower esophageal sphincter tone. See discussion section for full details as to why we recommend first-line stenting over PCN placement. *Expert opinion*.

For obstetric patients that have been counseled by an obstetrician and recommended to have intra-operative fetal monitoring, stent placement should be performed in the proximity of specialties such as anesthesia, obstetrics, and neonatology. *Clinical principle*.

For patients with prior GU reconstruction (e.g., conduit, neobladder, transplant kidney), or a stone large enough to require future percutaneous nephrolithotomy, percutaneous nephrostomy (PCN) tube should be the first line intervention (Figure 3). Interventional radiology (IR) should be notified when attempting ureteral stenting so that the patient can be quickly treated by IR if stenting fails. Patients should be consented for both ureteral stent and PCN placement prior to attempted stent placement so IR can proceed with the PCN placement without requiring emergence from anesthesia for the patient to obtain another consent. If the patient refuses to have radiation with fluoroscopy, PCN placement under ultrasound access is an option. *Expert opinion*.

Obstetricians will determine whether intraoperative fetal monitoring is indicated. When appropriate, the OB team will obtain informed consent for possible emergency cesarean delivery, arrange for delivery equipment, and should notify the neonatal intensive care unit. *Expert opinion*.

#### Recommendation 7

When placed, ureteral stents should be exchanged every 4 weeks until definitive management is performed. *Strong recommendation, Evidence Strength C*.

#### Recommendation 8

If conservative management fails, ureteroscopy with laser lithotripsy should be offered as a first-line treatment in non-complex scenarios (refer to interventional flowchart/discussion section for definitions of complexity–Figure 3). *Strong recommendation, Evidence Strength B*.

#### Recommendation 9

Ureteroscopy during the third trimester may be associated with higher rates of pre-term labor, however it should not be excluded as a treatment option. When clinically prudent, the decision to proceed with ureteroscopy should be determined after a discussion between the patient, urology and obstetrics teams occur. The third trimester is from 28 to 40 weeks. Once at 32 weeks gestation, risks to the fetus quickly decline as the pregnancy progresses with similar long-term outcomes as a full-term neonate. Betamethasone for fetal lung maturation may be administered as well as magnesium sulfate for fetal neuroprotection (12). A neonatology consult should be considered. *Moderate recommendation, Evidence Strength B*.

#### Recommendation 10

If appropriate, neuraxial anesthesia (spinal, epidural, or combined spinal-epidural) is preferred over general anesthesia in obstetric patients given that the physiologic changes of pregnancy increase the incidence of aspiration and difficult airway management. Neuraxial anesthesia also limits fetal exposure to anesthetic agents and medications. However, these considerations do not preclude a patient from receiving general anesthesia when necessary and there is no clear evidence that it poses a greater risk to the fetus. The potential for difficult airway management and aspiration should be considered and anticipated when planning for any type of anesthetic. *Moderate recommendation, Evidence Strength B*.

#### Recommendation 11

Patients discharged to home with indwelling ureteral stents/PCN tubes or residual stones should have established outpatient follow-up with a urology provider and their obstetric provider. Definitive treatment with ureteroscopy can then be offered as an outpatient. *Expert Opinion*.

## Discussion

In this section we discuss the rationale and evidence behind our guideline statements. Furthermore, several flowcharts are also included that provide an easy-to-follow flowchart to assist with diagnostic and interventional management. Figure 1 summarizes the initial evaluation of a patient presenting with nephrolithiasis during pregnancy. Figure 2 displays the flowchart for when diagnostic uncertainty exists after obtaining the initial work up. Figure 3 provides the schema for interventional management.

### Part 1: Initial Work Up/Imaging Guidelines

#### Renal Bladder Ultrasound

RBUS is the preferred first-line imaging modality for the gravid patient presenting with renal colic and suspected obstructing nephrolithiasis given it is non-invasive, widely available, lacks ionizing radiation, and is cost effective (13). Although a useful screening test, the limitations of RBUS should be discussed with the patient. Ultrasound has limited sensitivity (34–95%) and specificity (34%) for obstructive nephrolithiasis in pregnancy (14). Presence of hydronephrosis alone is often not enough to distinguish obstructing urolithiasis from physiologic hydronephrosis. In up to 90% of pregnancies, physiologic hydronephrosis can be present (15).

If not included in the initial report, other findings to discuss with radiology that can improve the discriminative capability of RBUS include the presence/absence of ureteral jets or presence of elevated resistive indices (RI). To prevent false positives, ureteral jets are best evaluated with a well hydrated patient while they are lying in the contralateral decubitus position (14). Absence of a ureteral jet unilaterally has a sensitivity of up to 100% and specificity of 91% (16). Evaluation of RI can help improve specificity of ultrasound for obstructing nephrolithiasis. Unilateral RI > 0.70 are indicative of acute obstruction within the last 6 h (sensitivity 45%, specificity 91%) and are not typically elevated with hydronephrosis of pregnancy (17). Similarly, a difference in RI between the normal and obstructed kidney of 0.06 has been shown to be 95% sensitive and 100% specific for acute ureteral obstruction in pregnancy (17). If suspecting a distal ureteral stone based on symptoms, transvaginal imaging has also been shown to be more specific for stones located in the distal ureter (18).

#### CT and Second-Line Imaging Studies

Due to the very low risk of adverse effects from exposure to ionizing radiation during low-dose stone CT, both the American College of Radiology (ACR) and the American College of Obstetrics and Gynecology (ACOG) support the use of abdominal/pelvic CT if medically necessary, even in the first trimester (9, 19). Thus, in scenarios where there is clinical decompensation of the patient and the diagnosis behind renal colic remains uncertain, CT should be considered as the next diagnostic study (Figure 2). For all patients, it is considered best practice to use as low as reasonably achievable (ALARA) ionizing radiation. This principle applies during pregnancy as well, as there are studies that suggest theoretical concerns of teratogenesis, cognitive impairment, or other fetal harm associated with using ionizing radiation in pregnancy. However, at the doses commonly prescribed for abdominal CT imaging, this risk is exceedingly low. Radiation doses common to abdominal CT imaging (<50 mGy) have not been associated with any cases of fetal harm (20). It is estimated that doses of 20 mGy increase the lifetime cancer risk to 0.8%, or 40 individuals out of 5,000 (Abdominal CT ranges from 1.5 to 35 mGy. Newer CT machines can achieve very low mGy). These risks are based upon data from animal studies, survivors of nuclear incidents, and cancer patients (9, 21).

Non-contrast CT has the highest sensitivity and specificity for detecting obstructing calculi (22). Presence of an obstructing stone on CT is also associated with highest rates of subsequent positive ureteroscopic findings (23). To adhere to the ALARA principle, low-dose NC-CT should be offered to further minimize radiation exposure. Consultation with a medical physicist (if available) can help to ensure that scans are protocoled to keep radiation doses to a minimum, while maintaining diagnostic accuracy. Consultation with medical physics should be strongly considered when the patient has already been exposed to multiple studies using ionizing radiation previously in the pregnancy.

If the clinical status of the patient does not warrant emergent diagnosis (i.e., patient is not in septic shock or in renal failure), repeat RBUS can be offered to the patient (Figure 2). Repeat ultrasound can occasionally identify migrating stones that were initially missed. If not performed on the initial ultrasound, maneuvers such as measuring ureteral jets, RIs, and transvaginal approaches can be used to augment the discriminative capability of ultrasound. This recommendation is based on local expert opinion, as there is a lack of high-quality data on the utility of repeat ultrasound imaging in obstetric patients.

Magnetic resonance imaging (MRI) confers no ionizing radiation and could be performed without contrast to assess for collecting system obstruction (Figure 2). A single shot fast spin-echo technique that produces heavily T2-weighted, high-resolution images, e.g., HASTE (HASTE is an imaging sequence trademarked by Siemens (Siemens AG, Munich, Germany) that stands for: Half-Fourier Acquisition Single-shot Turbo-spin Echo) or SS-FSE (Single-shot Fast Spin Echo): the same sequence on GE machines (General Electric Healthcare, Chicago, Illinois), at a higher field strength (1.5 or 3.0 Tesla), can depict collecting system dilatation and perinephric fluid with high specificity. MRI has poor sensitivity for depicting calculi, with only 50% of calculi seen at MRI when compared to CT (24). There is a lack of prospective data validating the utility of MRI. A recent prospective study demonstrated similar positive predictive value (PPV) (calculated using ureteroscopy as gold standard) between MRI and ultrasound (80 vs. 77%), but inferior PPV for MRI vs. CT (80 vs. 95.2%) (23). However, an MRI may not be able to be obtained as quickly as a CT in an emergent scenario (e.g., longer scan time and wait time), making it less useful in urgent situations. There are also theoretical concerns of tissue heating and hearing loss to the fetus when using MRI. Tissue heating can be increased with high specific absorption rate sequences like MRI HASTE/SS-FSE. However, there has been no data to suggest MRI causes significant temperature changes or risks to the fetus, even with the SS-FSE sequences. Tissue heating also decreases as distance increases from the radiofrequency pulses. Nevertheless, the International Commission on Non-Ionizing Radiation Protection (ICNIRP) recommends postponing elective MRI to after the first trimester because of the above theoretical risks (9, 25).

In conclusion, although the effect of ionizing radiation on the fetus from a medical imaging test is uncertain, the radiation dose from a single NC-CT study is very small and the risk to the fetus is considered to be very low. As such, the ACR and the ACOG support the use of abdominopelvic CT for suspected nephrolithiasis if medically necessary, even in the first trimester. In scenarios where the patient is clinically worsening and the diagnosis of renal colic needs to be determined with certainty, NC-CT should be considered as the next diagnostic study.

### Discussion Part 2: Intervention

#### Trial of Spontaneous Passage

It is estimated that 50–80% of stones will pass spontaneously during pregnancy (26, 27). Therefore, conservative management is a reasonable option as long as symptoms are manageable (26, 28, 29). Selective Alpha-1 blockers are category B drugs in pregnancy and thought to be safe (5). Although a small cohort, the safety of alpha1-blocker therapy with tamsulosin was demonstrated in one retrospective study, which showed no significant differences in maternal or fetal outcomes (11). Patients should have established follow up with an urologist to ensure that the stone passes. If conservative management fails, definitive stone treatment “i.e., ureteroscopy” should be offered to prevent long term damage to the obstructed renal unit.

#### Anesthetic Management

The ACOG Committee Opinion on non-obstetric surgery during pregnancy states “there is no evidence, when given in standard concentrations, that *in utero* exposure to anesthetics is associated with an increased risk of teratogenicity or fetal harm” (30). General and regional (spinal, epidural, and combined spinal-epidural) anesthesia can be safely administered during pregnancy when indicated. However, the physiologic changes to the respiratory and gastrointestinal system during pregnancy increase the risk of difficult airway management and aspiration. Therefore, when feasible, stent placement under regional anesthesia is preferred during pregnancy.

The selection of anesthetic technique for a given surgical procedure should be considered on an individual basis, however, the physiologic changes of pregnancy shift the relative risk and safety of general vs. neuraxial techniques (10). There is an increased risk of difficult airway management in obstetric patients secondary to airway changes including capillary engorgement and tissue friability, increased oxygen consumption, decreased functional residual capacity, rapid desaturation with apnea, and enlarged breasts that can make laryngoscopy challenging (31, 32). Gastrointestinal changes during pregnancy such as decreased lower esophageal sphincter tone and slowing of esophageal peristalsis and intestinal transit increase the risk of gastric aspiration (31, 32). Historically, concerns have been raised regarding neurotoxicity in the developing fetal brain based on animal studies of systemic anesthesia and this may favor regional techniques in the mother to reduce fetal exposure to general anesthetics (10, 33). Despite these concerns, there are circumstances that require general anesthesia for surgery in the obstetric patient and there are no human studies that have demonstrated teratogenicity from anesthetic agents when used in standard concentrations for <3 h (30). A single center, retrospective, case-control study from 2019 found that patients who received general anesthesia had a small increase in low birth weight in the newborn compared to those who received regional anesthesia, however, this association may simply reflect the patient's underlying condition rather than a direct effect of anesthesia (33). If the clinical condition of the patient and the procedure is amenable to neuraxial techniques, it should be considered to avoid fetal exposure to systemic anesthesia.

More important than the choice of technique is the need to maintain adequate control of hemodynamics and oxygenation throughout the course of the anesthetic. Care should be taken to avoid hypotension, hypoxia, hypercarbia, and hypocarbia (34). Maternal hypoxia results *in uteroplacental* insufficiency and can cause fetal hypoxemia, acidosis, and distress (34). Maternal ventilation should be maintained within the normal PaCO_2_ of pregnancy, between 30 and 32 mmHg to avoid uterine artery vasoconstriction and resultant fetal acidosis (34). Blood pressure should be maintained by ensuring adequate left uterine displacement “i.e., elevate the right hip 15 or more degrees, if the patient is < 20 weeks gestation or if the uterine fundus is at the level of the umbilicus, and with vasopressors as needed” (12, 18). Phenylephrine is the most commonly used agent to treat maternal hypotension (35).

Volatile anesthetic agents reduce uterine activity, however there is no data to suggest that this is beneficial in preventing preterm labor. Following surgery during pregnancy, the risk of preterm labor is increased and therefore if the fetus is viable, patients should be monitored post-operatively with tocographic and fetal heart rate monitoring per the obstetrician's recommendations (30).

In certain cases, PCN may be a better option. The rationale is: if IR can place the PCN under local anesthesia, ureteral stenting under regional or general anesthesia can be avoided with less systemic and hemodynamic effects. For example, in patients with impending septic shock, regional anesthesia may cause a precipitous decrease in blood pressure. Indeed, septic shock is a contraindication to regional anesthesia. Therefore, for patients with sepsis who are in the first trimester, PCN placement may be preferable if IR can place the nephrostomy tube urgently with local anesthesia and sedation.

However, PCN placement after the first trimester has several problematic issues. First, the patient may be at an increased risk for aspiration and many anesthesiologists will opt for general anesthesia, rather than sedation (34). Second, the prone position may transmit pressure from the gravid uterus onto the great vessels leading to decreased uterine perfusion with subsequent reduced fetal perfusion and maternal hypotension. Third, fetal monitoring is difficult in the prone position. Thus, in most cases ureteral stenting is safer for the obstetric patient and the fetus. However, if IR PCN must be performed, bolsters should be carefully positioned under the obstetric patient to ensure the abdomen is not flat on the operating room table. Furthermore, interventional radiologists can also place PCNs in a semi-prone/oblique position if a complete prone position is not possible.

#### Collecting System Decompression: Ureteral Stenting vs. Percutaneous Nephrostomy Tube Placement

Both ureteral stent and percutaneous nephrostomy tube placement are highly successful procedures with no high quality evidence in the literature to recommend one modality over the other (36). However, we recommend an attempt at ureteral stent placement as the initial management option. In one retrospective review of a large database of 3,904 obstetric patients with nephrolithiasis, those who underwent PCN placement had a preterm delivery rate of 19.6% from a baseline of 9.1% for women who had stones managed conservatively. Those who underwent ureteroscopy or ureteral stenting had a preterm delivery rate of 11.2% (37). Therefore, there may be a higher preterm delivery rate associated with PCN tube placement.

Other considerations of intervention include quality of life. Ureteral stents are associated with pain, encrustation, infection, and bladder irritability. Similarly, PCN tubes are associated with pain, infection, tube dislodgement, tube obstruction by debris, and bleeding (38). In one case series, half of the women receiving PCN tubes had to undergo tube exchanges/replacements due to occlusion from debris/tube dislodgement (18). One potential advantage of PCN tubes is that it can potentially be placed by IR under ultrasound guidance without use of fluoroscopy. Tips to minimize fluoroscopy use during stent placement are provided in the Box 1 (39).

Box 1 **Technical considerations for retrograde ureteral stent placement.**

- Place a bump underneath the patient's right side so that the uterus is displaced to the left, this relieves pressure on the IVC and helps stabilize blood pressures^*^
- Minimize use of fluoroscopy
  – Use spot fluoroscopy rather than live imaging
  – Decrease the number of shots per second (pre-set is 15 frames/second, but can go as low as 3 frames/second)
  – Use tactile feel to confirm placement of wire
  – Use ultrasound to confirm proximal stent curl

^*^Should be performed after 20 weeks gestation or if uterine fundus is at the umbilicus. Achieve at least a 15-degree tilt

Regardless of the tube type, due to gestational hyperfiltration and resulting hyperuricosuria and hypercalciuria, there is a higher rate of tube encrustation during pregnancy and expedited follow up must be scheduled to ensure timely removal/exchange (38). Thus, we recommend exchanging ureteral stents/PCNs every 4 weeks until definitive stone management.

#### Role of Primary Ureteroscopy

With advancements in endoscopic and laser technology, virtually any aspect of the upper and lower urinary system is accessible to treatment. The ureters are naturally dilated in pregnancy, which facilitates treatment. As discussed previously, drainage tubes are subject to infection and encrustation and require frequent exchanges. Ureteroscopy offers the potential for treating the stone in one session with the theoretical benefit of reduced exposure to anesthetics and avoiding side effects of indwelling tubes as long as the urine is not infected.

Multiple studies have shown that ureteroscopy in pregnancy appears to be safe with complication rates that are comparable to non-obstetric patients (40–43). There were no significant differences in urinary tract infection or ureteral injury. In a retrospective review of 112 women with stones during pregnancy over 12 years, there were no obstetric related complications after ureteroscopy and laser lithotripsy (29 women). In comparison, 42.1% of the women who underwent stent placement required early induction of labor at 38 weeks gestation. 10.9% of the patients who had stents experienced preterm labor within 24 h of stent placement (44).

The ACOG suggests that because of the consistent observation of higher incidence of preterm labor in the third trimester, any surgical intervention is recommended to occur during the second trimester if possible (30). This recommendation is extrapolated from general surgery literature (urgent abdominal surgery) (45, 46) with the limitation that these studies did not include endourologic surgery.

#### Multidisciplinary Operative Intervention

It is paramount that a multi-disciplinary approach is taken with any surgical intervention during pregnancy. At our institution, an emergent ureteral stent for an obstetric patient with a viable fetus (determined after an OB and neonatology consult) is performed at the women and children's hospital, given the proximity to the Neonatal Intensive Care Unit and OB staff in the event an emergent cesarean delivery of a preterm infant care is necessary. Obstetric and neonatal intensive team members are present in the operating room during the pre-induction verification for situational awareness and to ensure necessary medications, surgical and resuscitative equipment are available. The obstetric team will determine if continuous fetal monitoring during the procedure is necessary and will remain readily available to perform a cesarean delivery. Interventional radiology is also notified of a possible pending procedure if the ureteral stent placement fails.

## Conclusion

The establishment of multi-disciplinary, standardized guidelines for the management of nephrolithiasis during pregnancy has improved and streamlined the care for obstetric patients with nephrolithiasis at our institution. In circumstances where a RBUS demonstrates hydronephrosis but fails to demonstrate an obstructing ureteral stone, urologists may hesitate to order a NC-CT due to concern of fetal radiation. We provide evidence-based guidelines that demonstrate a NC-CT is safe and can be used when diagnostic uncertainty exists. Furthermore, in cases of septic obstructing stones we recommend ureteral stent placement as the preferred first line intervention. Ultimately, a multi-disciplinary approach for the management of obstetric patients with nephrolithiasis, in conjunction with the availability of subspecialty expertise is critical. These guidelines may be modified to fit the needs of the patient and surgical staff in their local environment.

## Author Contributions

ML, MF, EN, SC, and KG: project development, data collection, manuscript writing, and data analysis. CV, MB, JK, JE, JS, SA, AK, and WR: manuscript writing. All authors contributed to the article and approved the submitted version.

## Conflict of Interest

AK is a consultant for Ambu, Boston Scientific, Lumenis, and Virtuoso Surgical. She is a board member of Sonomotion. WR is a consultant for Boston Scientific. KG is a consultant for Boston Scientific, Lumenis, Coloplast, Olympus, and Karl Storz. The remaining authors declare that the research was conducted in the absence of any commercial or financial relationships that could be construed as a potential conflict of interest.

## Publisher's Note

All claims expressed in this article are solely those of the authors and do not necessarily represent those of their affiliated organizations, or those of the publisher, the editors and the reviewers. Any product that may be evaluated in this article, or claim that may be made by its manufacturer, is not guaranteed or endorsed by the publisher.
