# Supplementary material for: Management of Nephrolithiasis in Pregnancy: Multi-Disciplinary Guidelines From an Academic Medical Center
Source: Front Surg. 2021 Dec 22;8:796876. doi: 10.3389/fsurg.2021.796876 (PMC8751485; doi:10.3389/fsurg.2021.796876)
Supplement: Supplementary file 1 [file Data_Sheet_1.docx]

1. ACOG Practice Bulletin No. 211: Critical Care in Pregnancy. Obstetrics & Gynecology. 2019;133(5):e303-e319. doi:10.1097/aog.0000000000003241

2. 723 ACON. Guidelines for Diagnostic Imaging During Pregnancy and Lactation. Obstet Gynecol. 2017;127:e75-80.

3. 775 ACO. Nonobstetric Surgery During Pregnancy. Obstetrics & Gynecology. 2019;133(4):e285-286.

4. Afolabi BB LF. Regional versus general anaesthesia for caesarean section. The Cochrane database of systematic reviews 2012;10(Cd004350)

5. Assimos D KA, Miller NL et al. Surgical management of stones: American Urological Association/Endourological Society Guideline. J Urol. 2016;196(II)

6. Bailey G, Vaughan L, Rose C, Krambeck A. Perinatal Outcomes with Tamsulosin Therapy for Symptomatic Urolithiasis. J Urol. Jan 2016;195(1):99-103. doi:10.1016/j.juro.2015.06.097

7. Bleeser T, Van Der Veeken L, Fieuws S, et al. Effects of general anaesthesia during pregnancy on neurocognitive development of the fetus: a systematic review and meta-analysis. Br J Anaesth. Jun 2021;126(6):1128-1140. doi:10.1016/j.bja.2021.02.026

8. Brian R. Matlaga AEK, James E. Lingeman. Surgical Management of Upper Urinary Tract Calculi. In: Wein AJK, Louis R; Partin, Alan W; Peters, Craig A, ed. Campbell Walsh Urology. 11 ed. Elsevier; 2016:1260-1290:chap 54.

9. Burgess KL, Gettman MT, Rangel LJ, Krambeck AE. Diagnosis of urolithiasis and rate of spontaneous passage during pregnancy. J Urol. Dec 2011;186(6):2280-4. doi:10.1016/j.juro.2011.07.103

10. Chen TT, Wang C, Ferrandino MN, et al. Radiation Exposure during the Evaluation and Management of Nephrolithiasis. J Urol. Oct 2015;194(4):878-85. doi:10.1016/j.juro.2015.04.118

11. Cluver C NN, Hofmeyer GJ, Hall DR. Maternal position during caesarean section for preventing maternal and neonatal complications. Cochrane Database Syst Rev. 2013;28(3)

12. Dauw CA, Simeon L, Alruwaily AF, et al. Contemporary Practice Patterns of Flexible Ureteroscopy for Treating Renal Stones: Results of a Worldwide Survey. J Endourol. Nov 2015;29(11):1221-30. doi:10.1089/end.2015.0260

13. Devroe S, Bleeser T, Van de Velde M, et al. Anesthesia for non-obstetric surgery during pregnancy in a tertiary referral center: a 16-year retrospective, matched case-control, cohort study. Int J Obstet Anesth. Aug 2019;39:74-81. doi:10.1016/j.ijoa.2019.01.006

14. Deyoe LA CJ, Breslaw BH, et al. New techniques of ultrasound and color Doppler in the prospective evaluation of acute renal obstruction. Do they replace the intravenous urogram? . Abdom Imaging. 1995;20:58-63.

15. Drescher M, Blackwell RH, Patel PM, Kuo PC, Turk TMT, Baldea KG. Antepartum nephrolithiasis and the risk of preterm delivery. Urolithiasis. Oct 27 2018;doi:10.1007/s00240-018-1085-3

16. E G. Principles and practices of obstetric airway management. Anesthesiology clinics. 2008;26:109-25.

17. Eisner BH, Dretler SP. Use of the Stone Cone for prevention of calculus retropulsion during holmium:YAG laser lithotripsy: case series and review of the literature. Urol Int. 2009;82(3):356-60. doi:10.1159/000209372

18. Evans HJ WT. The management of urinary calculi in pregnancy. Current opinion in Urology. 2001;1:379-384.

19. Fulgham PF, Assimos DG, Pearle MS, Preminger GM. Clinical effectiveness protocols for imaging in the management of ureteral calculous disease: AUA technology assessment. J Urol. Apr 2013;189(4):1203-13. doi:10.1016/j.juro.2012.10.031

20. Goldfarb S HH, Heilbrun ME, Heller MT, Nikolaidis P, Preminger GM, et al. . ACR Appropriateness Criteria: Acute Onset Flank Pain-Suspicion of Stone Disease (Urolithiasis). ACR. 2015;

21. Hawkins JL CJ, Palmer SK, Gibbs CP, Callaghan WM. Anesthesia-related maternal mortality in the United States: 1979-2002. Obstetrics and gynecology 2011;117:69-74.

22. Hawkins JL KL, Palmer SK, Gibbs CP. Anesthesia-related deaths during obstetric delivery in the United States, 1979-1990. Anesthesiology. 1997;86(86):277-84.

23. HN GEaW. Renal calculi in pregnancy. Br J Uro. 1997;80(1):4-9.

24. Hoscan MB, Ekinci M, Tunckiran A, Oksay T, Ozorak A, Ozkardes H. Management of symptomatic ureteral calculi complicating pregnancy. Urology. Nov 2012;80(5):1011-4. doi:10.1016/j.urology.2012.04.039

25. Hsu L, Li H, Pucheril D, et al. Use of percutaneous nephrostomy and ureteral stenting in management of ureteral obstruction. World J Nephrol. Mar 6 2016;5(2):172-81. doi:10.5527/wjn.v5.i2.172

26. Jarrard DJ GG, Lyon ES. . Management of acute ureteral obstruction in pregnancy utilizing ultrasound guided placement of ureteral stents. Urology. 1993;42:263-267.

27. Johnson EB, Krambeck AE, White WM, et al. Obstetric complications of ureteroscopy during pregnancy. J Urol. Jul 2012;188(1):151-4. doi:10.1016/j.juro.2012.02.2566

28. Laing KA, Lam TB, McClinton S, Cohen NP, Traxer O, Somani BK. Outcomes of ureteroscopy for stone disease in pregnancy: results from a systematic review of the literature. Urol Int. 2012;89(4):380-6. doi:10.1159/000343732

29. Leffert L, Butwick A, Carvalho B, et al. The Society for Obstetric Anesthesia and Perinatology Consensus Statement on the Anesthetic Management of Pregnant and Postpartum Women Receiving Thromboprophylaxis or Higher Dose Anticoagulants. Anesthesia & Analgesia. 2018;126(3):928-944. doi:10.1213/ane.0000000000002530

30. Lloyd GL, Lim A, Hamoui N, Nakada SY, Kielb SJ. The Use of Medical Expulsive Therapy During Pregnancy: A Worldwide Perspective Among Experts. J Endourol. Mar 2016;30(3):354-8. doi:10.1089/end.2015.0587

31. Matlaga BR, Chew B, Eisner B, et al. Ureteroscopic Laser Lithotripsy: A Review of Dusting vs Fragmentation with Extraction. J Endourol. Jan 2018;32(1):1-6. doi:10.1089/end.2017.0641

32. McCollough CH SB, Atwell TD, et al. Radiation exposure and pregnancy: when should we be concerned? Radiographics. 2007;27(4):909-917.

33. Mhyre JM HD. The unanticipated difficult intubation in obstetrics. Anesthesia and analgesia. 2011;112:648-652.

34. Mullins JK, Semins MJ, Hyams ES, Bohlman ME, Matlaga BR. Half Fourier single-shot turbo spin-echo magnetic resonance urography for the evaluation of suspected renal colic in pregnancy. Urology. Jun 2012;79(6):1252-5. doi:10.1016/j.urology.2011.12.016

35. Ni Mhuireachtaigh R, O'Gorman DA. Anesthesia in pregnant patients for nonobstetric surgery. J Clin Anesth. Feb 2006;18(1):60-6. doi:10.1016/j.jclinane.2004.11.009

36. Parulkar BG HT, Wollin MR et al. . Renal colic during pregnancy: a case for conservative treatment. J Urol. 1998;159:365.

37. Patel SJ RD, Katz DS, et al. Imaging the pregnant patient for nonobstetric conditions: algorithms and radiation dose considerations. Radiographics. 2007;27:1705-1722.

38. Radiology ACo. ACR–SPR PRACTICE PARAMETER FOR IMAGING PREGNANT OR POTENTIALLY PREGNANT ADOLESCENTS AND WOMEN WITH IONIZING RADIATION. ACR. 2018;

39. Rana AM, Aquil S, Khawaja AM. Semirigid ureteroscopy and pneumatic lithotripsy as definitive management of obstructive ureteral calculi during pregnancy. Urology. May 2009;73(5):964-7. doi:10.1016/j.urology.2008.12.054

40. Reitman E, Flood P. Anaesthetic considerations for non-obstetric surgery during pregnancy. Br J Anaesth. Dec 2011;107 Suppl 1:i72-8. doi:10.1093/bja/aer343

41. Rivera ME, McAlvany KL, Brinton TS, Gettman MT, Krambeck AE. Anesthetic exposure in the treatment of symptomatic urinary calculi in pregnant women. Urology. Dec 2014;84(6):1275-8. doi:10.1016/j.urology.2014.07.007

42. Semins MJ, Feng Z, Trock B, Bohlman M, Hosek W, Matlaga BR. Evaluation of acute renal colic: a comparison of non-contrast CT versus 3-T non-contrast HASTE MR urography. Urolithiasis. Feb 2013;41(1):43-6. doi:10.1007/s00240-012-0525-8

43. Semins MJ, Matlaga BR. Kidney stones during pregnancy. Nat Rev Urol. Mar 2014;11(3):163-8. doi:10.1038/nrurol.2014.17

44. Semins MJ, Trock BJ, Matlaga BR. The safety of ureteroscopy during pregnancy: a systematic review and meta-analysis. J Urol. Jan 2009;181(1):139-43. doi:10.1016/j.juro.2008.09.029

45. Shokeir AA MMaAM. Renal colic in pregnant women: role of renal resistive index. Urology. 2000;55:344-347.

46. Stothers L LL. Renal colic in pregnancy. J Urol. 1992;148(5)(1):1383-1387.

47. Swartz MA L-RM, Simon D, Wright JL, Porter MP. Admission for Nephrolithiasis in Pregnancy and Risk of Adverse Birth Outcomes. Obstet Gynecol. 2007;109:50-=:1099-1104.

48. Tirada N DD, Khati NJ, et al. Imaging Pregnant and Lactating Patients. RadioGraphics. 2015;35:1751-1765.

49. Upadhyay A, Stanten S, Kazantsev G, Horoupian R, Stanten A. Laparoscopic management of a nonobstetric emergency in the third trimester of pregnancy. Surg Endosc. Aug 2007;21(8):1344-8. doi:10.1007/s00464-006-9104-9

50. Van De Velde M DBF. 2007. Minerva Anestesiol. Anesthesia for non-obstetric surgery in the pregnant patient;73(4):235-240.

51. Visser BC GR, Mulvihill KK, Mulvihill SJ. Safety and Timing of Nonobstetric Abdominal Surgery in Pregnancy. Digestive Surgery. 2001;18:409-417.

52. Wang PI, Chong ST, Kielar AZ, et al. Imaging of pregnant and lactating patients: part 1, evidence-based review and recommendations. AJR Am J Roentgenol. Apr 2012;198(4):778-84. doi:10.2214/AJR.11.7405

53. Watterson JD GA, Beiko DT, Nott L, Wollin TA, Razvi H, Denstedt JD. Ureteroscopy and Holmium:YAG Laser Lithotripsy: An Emerging Definitive Management Strategy for Symptomatic Ureteral Calculi in Pregnancy. Urology. 2002;60(3):383-387.

54. White WM, Johnson EB, Zite NB, et al. Predictive value of current imaging modalities for the detection of urolithiasis during pregnancy: a multicenter, longitudinal study. J Urol. Mar 2013;189(3):931-4. doi:10.1016/j.juro.2012.09.076
